# Characteristics of Cerebral Autoregulation After Mechanical Thrombectomy and Its Relationship With Prognosis

**DOI:** 10.1111/cns.70323

**Published:** 2025-03-04

**Authors:** Yang Qu, Menglu Cong, Jia Liu, Pan‐Deng Zhang, Zi‐Duo Shen, Han Zhang, Yingying Sun, Hongjing Zhu, Chao Li, Peng Zhang, Junlei Chang, Kejia Zhang, Jiaxin Ren, Hang Jin, Xin Sun, Yi Yang, Zhen‐Ni Guo

**Affiliations:** ^1^ Stroke Center, Department of Neurology The First Hospital of Jilin University Changchun China; ^2^ Laboratory for Engineering and Scientific Computing, Institute of Advanced Computing and Digital Engineering, Shenzhen Institute of Advanced Technology Chinese Academy of Sciences Shenzhen China; ^3^ Center for Protein and Cell‐Based Drugs, Institute of Biomedicine and Biotechnology, Shenzhen Institute of Advanced Technology Chinese Academy of Sciences Shenzhen China; ^4^ Neuroscience Research Center, Department of Neurology The First Hospital of Jilin University Changchun China

**Keywords:** cerebral autoregulation, collateral circulation, mechanical thrombectomy, prognosis, revascularization, stroke

## Abstract

**Aims:**

To investigate the characteristics of dynamic cerebral autoregulation (dCA) in patients after mechanical thrombectomy (MT) and the relationship between dCA and prognosis.

**Methods:**

In this prospective study, 89 and 158 patients were enrolled in the MT and non‐MT groups, respectively. Both groups underwent dCA measurements within 3 days after stroke. The transfer function parameter, phase difference (PD), and gain were used to quantify dCA. A favorable outcome was defined as a modified Rankin Scale score ≤ 2 at 90 days. The collateral score was used to reflect the collateral reserve capacity.

**Results:**

MT was associated with better PD in both the affected and unaffected sides. In the MT group, the PD of the affected side was an independent predictor of favorable outcomes (odds ratio [OR] 0.927, 95% confidence interval [CI] 0.885–0.970; *p* < 0.001). The area under the receiver operating characteristic curve for predicting favorable outcomes of the PD on the affected side was 0.759 (95% CI, 0.654–0.864; *p* < 0.001). Further, good collaterals were independently associated with better PD.

**Conclusions:**

MT has a positive effect on dCA during the acute phase of stroke. For patients undergoing MT, dCA is a reliable indicator for predicting prognosis and may be an intervention target to improve outcomes.

## Graphical abstract

MT has a positive effect on dynamic cerebral autoregulation (dCA) at the acute phase of stroke. For MT patients, dCA is a reliable indicator for predicting the prognosis of patients and may be an intervention target to improve outcomes.

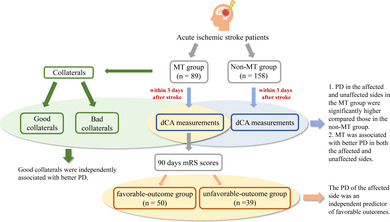

## 1 Introduction

For acute ischemic stroke with large vessel occlusion, mechanical thrombectomy (MT) is the recommended treatment for patients with limited signs of early ischemic changes on neuroimaging [1, 2]; however, patient responses to MT vary greatly. Therefore, it is imperative to find reliable indicators to predict prognosis after MT and to investigate possible intervention targets to improve the prognosis of these patients.

Stable hemodynamic [3] and intact blood–brain barrier function [4] are important contributors associated with clinical outcomes after MT, and dynamic cerebral autoregulation (dCA) is related to both of these [5, 6]. dCA can stabilize cerebral blood flow by regulating the contraction and relaxation of cerebral small vessels and microvessels, indicating that dCA can not only prevent hemorrhagic transformation caused by hyperperfusion after MT but also prevent infarct expansion caused by hypoperfusion [6]. In addition, dCA can alleviate blood–brain barrier leakage, reducing the amount of circulating inflammatory factors that infiltrate the brain to activate the glia [5]. Thus, dCA may be an important predictor of the prognosis for patients after MT. Theoretically, vascular recanalization after MT can improve the damage to dCA caused by a thrombus, but this does not necessarily occur in practice. First, MT may cause direct arterial damage [7], and second, the patients have a poor vascular foundation and most of them develop infarcts, all of which can affect dCA. Therefore, the characteristics of dCA after MT remain unclear. Furthermore, whether dCA can be used to predict the prognosis of patients with MT or serve as a possible intervention target to improve patient outcomes is currently unknown. In addition, previous studies have shown that good collaterals play an important role in stabilizing hemodynamics in patients with ischemic stroke [8, 9], but the relationship between collateral circulation and dCA in patients after MT is unclear.

Therefore, the aim of this study was to clarify the characteristics and roles of dCA in patients after MT by (1) investigating differences in dCA between patients with acute stroke who underwent MT and those who did not, (2) exploring the relationship between dCA and the prognosis of MT patients, and (3) exploring the effects of the collateral circulation on dCA in MT patients.

## 2 Materials and Methods

This prospective observational study followed the “Strengthening the Reporting of Observational Studies in Epidemiology (STROBE)” guidelines and was approved by the Ethics Committee of The First Hospital of Jilin University (2016‐294). Written informed consent was obtained from all participants, who had the right to withdraw from the study at any point. All procedures were carried out in accordance with the Declaration of Helsinki.

### 2.1 Participants and Study Design

We enrolled consecutive patients with ischemic stroke (within 24 h of stroke onset and not receiving intravenous thrombolysis) with large vessel occlusion from September 2016 to March 2022 at The First Hospital of Jilin University. Large vessel occlusion was defined as computed tomography angiography‐ or digital subtraction angiography (DSA)‐confirmed anterior or posterior circulation large vessel occlusion, involving the internal carotid artery, middle cerebral artery, basilar artery, or vertebral artery; unilateral tandem occlusion; or unilateral multi‐vessel occlusion.

Patients who underwent MT and met the following criteria were included in the MT group. Inclusion criteria: (a) age ≥ 18 years, male or female; (b) modified Rankin Scale (mRS) scores ≤ 2 before disease onset; (c) successful recanalization [thrombolysis in cerebral infarction score of ≥ 2b] [10] after MT; and (d) signed informed consent. We excluded patients (a) with myocardial infarction, heart failure, severe anemia, hyperthyroidism, or other confounders that influence cerebral hemodynamics; (b) in whom affected and unaffected sides were indistinguishable; (c) who could not cooperate with dCA monitoring; and (d) who had insufficient bilateral temporal bone windows for insonation of the middle cerebral artery.

Patients who did not undergo MT and met the following criteria were included in the non‐MT group. Inclusion criteria: (a) age ≥ 18 years, male or female; (b) mRS scores ≤ 2 before disease onset; and (c) signed informed consent. The exclusion criteria were the same as those for the MT group. Additionally, during dCA data analysis, data that could not be analyzed due to a substandard coherence function were excluded in both groups.

Affected and unaffected sides were distinguished according to symptoms, infarct location, and occluded arteries. Symptoms and infarct location were the leading criteria for patients with basal artery occlusion. Patients in the MT group underwent dCA measurements within 3 days after MT, while those in the non‐MT group underwent dCA at the same time as those in the MT group. Clinical outcomes were assessed using mRS scores 90 days after ictus via a telephone interview with participants or their relatives by an examiner who was blinded to the dCA results. Favorable outcomes were defined by an mRS score ≤ 2 at 90 days. The collateral score was used to reflect the collateral reserve capacity [11]. This DSA‐based score was assessed using the American Society of Interventional and Therapeutic Neuroradiology/Society of Interventional Radiology (ASITN/SIR) collateral flow grading system [12], with scores on a 5‐point scale (0–4). In this study, good collaterals were characterized as a score of 3 or 4 [13, 14].

### 2.2 dCA Evaluation

After surgery, the patients were transferred to the neurological intensive care unit. Only patients who met the criteria for exiting the intensive care unit, as assessed by surgeons, were evaluated for dCA in a dedicated room with controlled levels of ambient noise and temperature ranging from 20°C to 24°C, as previously reported [6, 15]. All patients underwent dCA measurements within 3 days after stroke onset.

Before the measurement, the patients were instructed to relax in a supine position for 10 min. Then, heart rate and blood pressure (assessed using an automatic blood pressure monitor [Omron 711, Japan]) were measured at the brachial artery, while the National Institutes of Health Stroke Scale (NIHSS) score was determined at the same time. Two 2‐MHz probes were fixed with a customized head frame at the bilateral temporal bone window at a depth of 45–60 mm, and continuous bilateral middle cerebral artery blood flow velocity (measured using transcranial Doppler [MultiDop X4; DWL, Sipplingen, Germany]) was recorded for approximately 10 min. Simultaneously, arterial blood pressure (ABP) at the fingertip of the middle finger (measured using a servo‐controlled plethysmograph [Finometer Model 1; FMS, Netherlands]) was recorded. End‐tidal CO_2_ was measured using a capnograph (MultiDop X4; DWL, Sipplingen, Germany) with a face mask attached to the nasal cannula.

The dynamic relationship between continuous bilateral middle cerebral artery blood flow velocity and fingertip ABP was assessed via transfer function analysis (TFA) using MATLAB (MathWorks; Natick, MA, USA) [16, 17]. A cross‐correlation function between ABP and cerebral blood flow velocity (CBFV) was used to align the data to eliminate potential time lags. A third‐order Butterworth anti‐aliasing low‐pass filter with a cutoff frequency of 0.5 Hz was applied to down‐sample the data to 1 Hz. Welch's method was employed to estimate the autospectrum of the ABP, $S_{\mathrm{xx}}\;(\;f\;)$, and the cross‐spectrum of ABP and CBFV, $S_{\mathrm{xy}}\;(\;f\;)$, in the frequency domain by averaging the periodograms of the down‐sampled ABP and CBFV with 50% overlapping hamming windows of 90 s in length. The transfer function, H(f), was then deviated as:

(1) $$H(\;f\;)=\frac{S_{\mathrm{xy}}(\;f\;)}{S_{\mathrm{xx}}\;(\;f\;)}$$

The gain and phase difference (PD) were then derived respectively from Equation (1) using Equations (2) and (3):

(2) $$\left|H(\;f\;)\right|=\sqrt{\left\{{\left|H_{R}(\;f\;)\right|}^{2}+{\left|H_{I}(\;f\;)\right|}^{2}\right\}}$$

(3) $$\theta =\tan^{-1}\left[H_{I}(\;f\;)/H_{R}(\;f\;)\right]$$

where *R* and *I* denote the real and imaginary parts of the transfer function, respectively. The parameters derived from the TFA within a low‐frequency range (0.06–0.12 Hz) included PD, gain, and the coherence function. Generally, a low PD at a low‐frequency band indicates impairment of autoregulation because it suggests that the CBFV follows the changes in ABP with a short delay. In addition, high gain at the same frequency band is an indicator of compromised autoregulation for passively transferring the amplitude of the ABP to the CBFV. The coherence function was used to test the linearity of CBFV and ABP values, and a critical coherence value of 0.34 (number of windows: 5; critical value of coherence: 5%) was selected as the threshold to establish the validity of the TFA estimates [16, 17]. Previous studies have verified that the PD is a much more sensitive component than the gain; therefore, our study mainly focused on the PD to reflect dCA function [18].

### 2.3 Statistical Analysis

Data were analyzed using the IBM SPSS Statistics version 19.0. The distribution of continuous variables was assessed using the Kolmogorov–Smirnov test. Normally distributed data were expressed as mean ± standard deviation and compared using the *t* test. Non‐normally distributed data were expressed as median (interquartile range) and compared using the Mann–Whitney *U* test. Categorical variables were expressed as absolute values and percentages and analyzed using the chi‐square test. Multivariate linear models were used to explore the influence of MT and collateral scores on dCA, and multivariate binary logistic regression analysis was used to explore the correlation between dCA and clinical outcomes. In both the linear and logistic analyses, four models were employed for the sensitivity analysis: Model 1 (unadjusted); Model 2 (adjusted for age, sex); Model 3 (adjusted for all variables) in Model 2 plus risk factors [including cigarette smoking, alcohol consumption, coronary heart disease, previous stroke, hypertension, and diabetes mellitus]; and Model 4 (adjusted for all variables in Model 3 plus clinical data). Clinical data in the multivariate linear analysis included fasting plasma glucose, infarct location, NIHSS score at admission, and systolic and diastolic blood pressure. In multivariate binary logistic analysis, clinical data included fasting plasma glucose, infarct location, the time from onset to femoral artery puncture, systolic and diastolic blood pressure, and the NIHSS score during dCA measurement. Only statistical results that were consistent in all four models were considered conclusive. A receiver operating characteristic (ROC) curve of the statistically significant variables associated with favorable outcomes was drawn. Statistical significance was set at a two‐tailed *p*‐value < 0.05.

## 3 Results

### 3.1 Demographic Information

**FIGURE 1:**
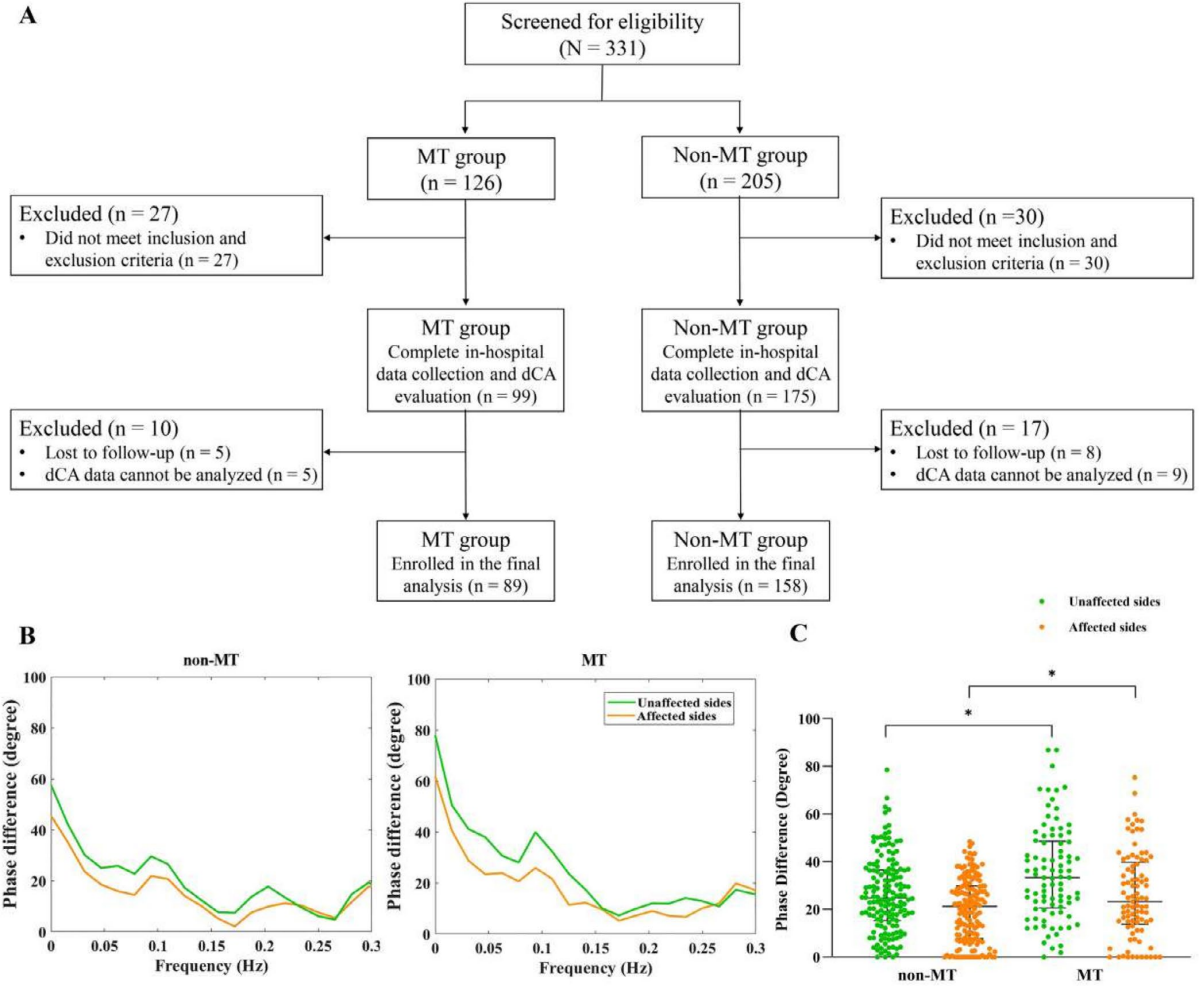
Flowcharts and phase differences in the non‐mechanical thrombectomy (MT) and MT groups. (A) Flowcharts of the study. (B) Phase differences (the main parameter of dynamic cerebral autoregulation) are derived from the transfer function analysis in the non‐MT and MT groups. (C) The statistical analysis of phase differences in the non‐MT and MT groups. *p < 0.05.

Overall, 331 patients were initially recruited, including 126 and 205 patients in the MT and non‐MT groups, respectively. In the MT group, the affected and unaffected sides could not be distinguished in 13 patients; seven patients could not cooperate with dCA monitoring; seven patients had insufficient bilateral temporal bone windows for insonation of the middle cerebral artery; five patients were lost to follow‐up; and dCA data of five patients could not be analyzed due to substandard coherence function. In the non‐MT group, the affected and unaffected sides could not be distinguished in 12 patients; six patients could not cooperate with dCA monitoring; 12 patients had insufficient bilateral temporal bone windows for insonation of the middle cerebral artery; eight patients were lost to follow‐up; and the dCA data of nine patients could not be analyzed due to substandard coherence function. Consequently, 89 and 158 patients from the MT and non‐MT groups, respectively, were included in the final analysis (Figure 1A). In the MT group, 50 patients had favorable outcomes and 39 had unfavorable outcomes. In the non‐MT group, 99 patients had favorable outcomes and 59 had unfavorable outcomes. The demographic and baseline characteristics of the patients included in both groups are presented in Table 1.

**TABLE 1:** Demographic characteristics and autoregulatory parameters in the mechanical thrombectomy (MT) and non‐MT groups.

	Non‐MT (*n* = 158)	MT (*n* = 89)	*t*/*Z*/*χ*2	*p*
Age (year)	59.09 ± 12.25	57.76 ± 11.26	−0.840	0.402
Sex (male, *n* [%])	150 (94.9%)	73 (82.0%)	10.823	**< 0.001**
Cigarette smoking, *n* (%)	98 (62.0%)	48 (53.9%)	1.543	0.214
Alcohol consumption, *n* (%)	69 (43.7%)	38 (42.7%)	0.022	0.882
Coronary heart disease, *n* (%)	18 (11.4%)	7 (7.9%)	0.779	0.378
Previous stroke, *n* (%)	35 (22.2%)	14 (15.7%)	1.476	0.224
Hypertension, *n* (%)	88 (55.7%)	49 (55.1%)	0.009	0.923
Diabetes mellitus, *n* (%)	36 (22.8%)	18 (20.2%)	0.218	0.640
Fasting plasma glucose, mmol/L	5.41 (4.94–6.31)	5.63 (5.05–7.24)	−1.642	0.101
Infract location			0.448	0.503
Anterior circulation, *n* (%)	109 (69.0%)	65 (73.0%)		
Posterior circulation, *n* (%)	49 (31.0%)	24 (27.0%)		
The time from onset to femoral Artery puncture (min)	—	360 (240–530)		
Favorable outcome	99 (62.7%)	50 (56.2%)	0.998	0.318
Admission data				
SBP, mmHg	143.43 ± 20.38	145.04 ± 24.98	0.550	0.583
DBP, mmHg	81.28 ± 13.00	82.40 ± 13.30	0.648	0.518
NIHSS score	4.00 (2.00–8.50)	10.0 (7.0–13.0)	−7.722	**< 0.001**
Collateral score				
0–2, *n* (%)	—	62 (69.7%)	—	—
3–4, *n* (%)	—	27 (30.3%)	—	—
Cerebral autoregulation				
Phase difference, degree				
Unaffected side	24.75 (15.28–36.51)	33.37 (20.60–48.59)	−3.392	**< 0.001**
Affected side	21.31 (7.74–29.85)	23.29 (13.82–39.81)	−2.128	**0.033**
Gain, %/mmHg				
Unaffected side	1.02 (0.76–1.38)	1.01 (0.76–1.42)	−0.217	0.828
Affected side	0.92 (0.66–1.15)	0.98 (0.70–1.31)	−1.449	0.147
Data during cerebral autoregulation measurements				
SBP	142.70 ± 20.52	136.42 ± 18.84	−2.380	**0.018**
DBP	80.92 ± 13.23	74.94 ± 12.07	−3.515	**< 0.001**
NIHSS score	3.00 (1.00–6.00)	4.0 (1.0–8.0)	−1.723	0.085
EtCO_2_, mmHg	40.89 ± 4.03	41.16 ± 4.47	0.488	0.626

*Note:* Favorable outcomes were defined by a modified Rankin Scale score ≤ 2 at 90 days. Age, SBP, DBP, and EtCO_2_ are expressed as mean ± standard deviation; fasting plasma glucose, NIHSS score, and cerebral autoregulation parameters are expressed as median (interquartile range); the other variables are expressed as *n* (%).

Abbreviations: DBP, diastolic blood pressure; EtCO, end‐tidal CO_2_; NIHSS, National Institutes of Health Stroke Scale; SBP, systolic blood pressure. Significance of bold values indicate statistically significant results in *p* < 0.05.

### 3.2 dCA Between MT and Non‐MT Groups

The PD of both affected and unaffected sides in the MT group within 3 days after MT was significantly higher than those in the non‐MT group (affected: 23.29 [13.82–39.81] vs. 21.31 [7.74–29.85], *p* = 0.033; unaffected: 33.37 [20.60–48.59] vs. 24.75 [15.28–36.51], *p* < 0.001). However, no difference was found in the gain of both sides between the two groups (Table 2, Figure 1B,C).

**TABLE 2:** The association between mechanical thrombectomy and autoregulatory parameters.

Mechanical thrombectomy	Phase difference			
	Unaffected side		Affected side	
	*β*	*p*	*β*	*p*
Model 1	8.932	< 0.001	5.845	**0.004**
Model 2	8.366	< 0.001	5.747	**0.006**
Model 3	8.712	< 0.001	5.888	**0.005**
Model 4	7.718	0.004	7.062	**0.003**

*Note:* Model 1 was unadjusted; Model 2 was adjusted for age and sex; Model 3 was adjusted for all variables in Model 2 plus risk factors (including cigarette smoking, alcohol consumption, coronary heart disease, previous stroke, hypertension and diabetes mellitus); Model 4 was adjusted for all variables in Model 3 plus clinical data (including fasting plasma glucose, infract location, and admission NIHSS score, SBP and DBP).

Abbreviations: DBP, diastolic blood pressure; NIHSS, National Institutes of Health Stroke Scale; SBP, systolic blood pressure. Significance of bold values indicate statistically significant results in *p* < 0.05.

In the linear models, MT was closely associated with PD of both the affected and unaffected sides; furthermore, the result was stable in the sensitivity analysis, indicating that MT has a positive effect on dCA in patients in the acute phase of ischemic stroke (Table 2).

### 3.3 Characteristics of dCA in the MT Group

#### 3.3.1 dCA In Favorable‐ and Unfavorable‐Outcome Groups

**FIGURE 2:**
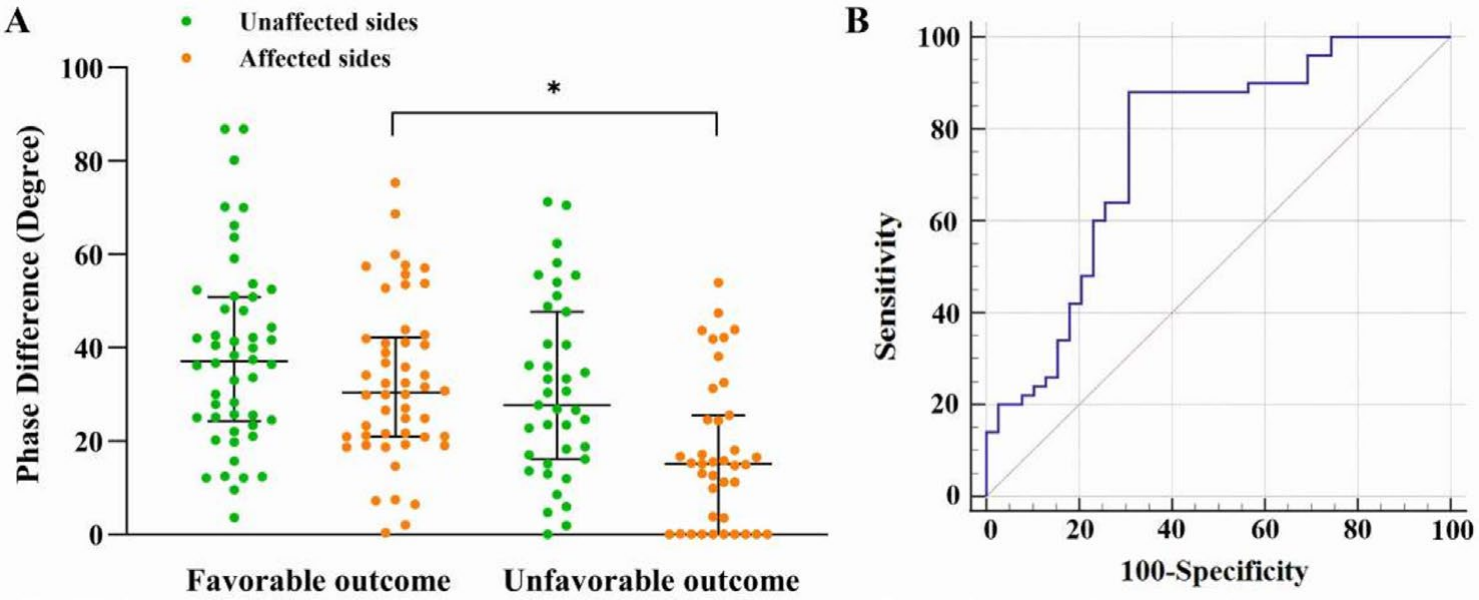
Phase difference and receiver operating characteristic curve in the mechanical thrombectomy (MT) group. (A) The difference in phase difference (the main parameter of dynamic cerebral autoregulation) between favorable‐ and unfavorable‐outcome patients in the MT group. (B) The receiver operating characteristic curve of phase difference of the affected sides predicting 3‐month favorable outcomes in the MT group. The area under the receiver operating characteristic curve for the prediction of favorable outcome was 0.759 (95% confidence interval, 0.612–0.886; *p* < 0.001), and the best cutoff point was 18.34° (sensitivity, 88.0%; specificity, 69.2%; positive predictive value, 78.6%; negative predictive value, 81.8%). *p < 0.05.

For patients with acute ischemic stroke who received MT, PD of the affected side in the favorable‐outcome group after surgery was significantly higher than that in the unfavorable‐outcome group (30.40 [20.93–42.19] vs. 15.15 [0.17–25.53], *p* < 0.001). There was no significant difference in PD of the unaffected side and gain of both sides between the two groups (Table 3 and Figure 2A).

**TABLE 3:** Demographic characteristics and autoregulatory parameters in the mechanical thrombectomy group with favorable and unfavorable outcomes.

	Favorable outcome (*n* = 50)	Unfavorable outcome (*n* = 39)	*t*/*Z*/*χ*2	*p*
Age (year)	57.58 ± 10.74	58.00 ± 12.04	−0.174	0.863
Sex (male, *n* [%])	43 (86.0%)	30 (76.9%)	1.224	0.269
Cigarette smoking, *n* (%)	28 (56.0%)	20 (51.3%)	0.196	0.658
Alcohol consumption, *n* (%)	24 (48.0%)	14 (35.9%)	1.312	0.252
Coronary heart disease, *n* (%)	3 (6.0%)	4 (10.3%)	0.548	0.459
Previous stroke, *n* (%)	8 (16.0%)	6 (15.4%)	0.006	0.937
Hypertension, *n* (%)	27 (54.0%)	22 (56.4%)	0.051	0.821
Diabetes mellitus, *n* (%)	6 (12.0%)	12 (30.8%)	4.784	**0.029**
Fasting plasma glucose, mmol/L	5.56 (5.05–6.50)	6.34 (5.07–8.55)	−1.733	0.083
Infract location			4.685	**0.031**
Anterior circulation, *n* (%)	41 (82.0%)	24 (61.5%)		
Posterior circulation, *n* (%)	9 (18.0%)	15 (38.5%)		
The time from onset to femoral artery puncture			1.350	0.245
Q1 (< 360 min)	28 (56.0%)	17 (43.6%)		
Q2 (≥ 360 min)	22 (44.0%)	22 (56.4%)		
Admission data				
SBP, mmHg	141.78 ± 24.65	149.23 ± 25.08	−1.404	0.164
DBP, mmHg	81.64 ± 12.64	83.38 ± 14.20	−0.612	0.542
NIHSS score	9.0 (5.0–13.0)	11.0 (7.0–15.0)	−1.601	0.109
Collateral score			5.041	**0.025**
0–2, *n* (%)	30 (60.0%)	32 (82.1%)		
3–4, *n* (%)	20 (40.0%)	7 (17.9%)		
Cerebral autoregulation				
Phase difference, degree				
Unaffected side	37.10 (24.29–50.91)	27.69 (16.09–47.73)	−1.679	0.093
Affected side	30.40 (20.93–42.19)	15.15 (0.17–25.53)	−4.178	**< 0.001**
Gain, %/mmHg				
Unaffected side	1.06 (0.76–1.44)	0.99 (0.75–1.25)	−0.947	0.344
Affected side	1.05 (0.74–1.44)	0.91 (0.64–1.25)	−1.629	0.103
Data during cerebral autoregulation measurements				
SBP	137.52 ± 18.42	135.00 ± 19.51	0.624	0.534
DBP	75.66 ± 11.72	74.03 ± 12.60	0.632	0.529
NIHSS score	3.0 (1.0–4.0)	6.5 (2.0–10.0)	−3.491	**< 0.001**
EtCO_2_, mmHg	40.64 ± 3.85	41.82 ± 5.12	−1.241	0.218

*Note:* Favorable outcomes were defined by a modified Rankin Scale score ≤ 2 at 90 days. Age, SBP, DBP, and EtCO_2_ are expressed as mean ± standard deviation; fasting plasma glucose, NIHSS score, and cerebral autoregulation parameters are expressed as median (interquartile range); the other variables are expressed as *n* (%).

Abbreviations: DBP, diastolic blood pressure; EtCO, end‐tidal CO_2_; NIHSS, National Institutes of Health Stroke Scale; SBP, systolic blood pressure. Significance of bold values indicate statistically significant results in *p* < 0.05.

#### 3.3.2 dCA and Prognosis

The results of the multivariate regression analysis showed that PD on the affected side was an independent predictor of a 90‐day good prognosis, and the predictive function was stable in the sensitivity analysis (Table 4).

**TABLE 4:** Multivariable regression analysis of the association between autoregulatory parameters after mechanical thrombectomy and favorable outcomes.

	Model 1		Model 2		Model 3		Model 4	
	OR (95% CI)	*p*	OR (95% CI)	*p*	OR (95% CI)	*p*	OR (95% CI)	*p*
Phase difference								
Unaffected side	0.980 (0.958–1.003)	0.084	0.978 (0.956–1.002)	0.067	0.975 (0.950–0.999)	**0.048**	0.974 (0.942–1.007)	0.115
Affected side	0.944 (0.916–0.974)	**< 0.001**	0.942 (0.913–0.972)	**< 0.001**	0.938 (0.907–0.971)	**< 0.001**	0.927 (0.885–0.970)	**< 0.001**

*Note:* Model 1 was unadjusted; Model 2 was adjusted for age and sex; Model 3 was adjusted for all variables in Model 2 plus risk factors (including cigarette smoking, alcohol consumption, coronary heart disease, previous stroke, hypertension and diabetes mellitus); Model 4 was adjusted for all variables in Model 3 plus clinical data (including fasting plasma glucose, infract location, the time from onset to femoral artery puncture, and SBP, DBP and NIHSS score during cerebral autoregulation measurement). Favorable outcomes were defined by a modified Rankin Scale score ≤ 2 at 90 days.

Abbreviations: CI, confidence interval; DBP, diastolic blood pressure; NIHSS, National Institutes of Health Stroke Scale; OR, odds ratio; SBP, systolic blood pressure. Significance of bold values indicate statistically significant results in *p* < 0.05.

The ROC curve of PD on the affected side is shown in Figure 2B. The area under the ROC curve for the prediction of a favorable outcome was 0.759 (95% confidence interval [CI], 0.654–0.864; *p* < 0.001), and the best cutoff point was 18.34° (sensitivity, 88.0%; specificity, 69.2%; positive predictive value, 78.6%; negative predictive value, 81.8%).

### 3.4 The Impact of the Collateral Score on dCA in the MT Group

For patients that underwent MT, good collaterals (*β* = 19.595; *p* < 0.001) were closely associated with better PD on the affected side. There was no significant association between PD on the unaffected side and gain on both sides with collateral levels (Table 5).

**TABLE 5:** The association between collateral score and phase difference after mechanical thrombectomy.

Good collaterals	Phase difference			
	Unaffected side		Affected side	
	*β*	*p*	*β*	*p*
Model 1	8.005	0.080	16.630	**< 0.001**
Model 2	8.924	0.056	17.282	**< 0.001**
Model 3	7.760	0.134	18.951	**< 0.001**
Model 4	7.929	0.151	19.595	**< 0.001**

*Note:* Model 1 was unadjusted; Model 2 was adjusted for age and sex; Model 3 was adjusted for all variables in Model 2 plus risk factors (including cigarette smoking, alcohol consumption, coronary heart disease, previous stroke, hypertension and diabetes mellitus); Model 4 was adjusted for all variables in Model 3 plus clinical data (including fasting plasma glucose, infract location, and admission NIHSS score, SBP and DBP).

Abbreviations: DBP, diastolic blood pressure; NIHSS, National Institutes of Health Stroke Scale; SBP, systolic blood pressure. Significance of bold values indicate statistically significant results in *p* < 0.05.

## 4 Discussion

In this study, we found that MT had a positive effect on dCA in the acute phase of stroke. PD of the affected side within 3 days after MT was an independent predictor of favorable outcomes. The area under the ROC curve for the prediction of favorable outcomes was 0.759, and the best cutoff point was 18.34°. Our study demonstrated that dCA is a reliable indicator for predicting the prognosis in patients with acute stroke after MT, and it may also be a specific intervention target to improve the prognosis of these patients. Furthermore, we demonstrated that good collaterals were associated with better dCA after MT.

The maintenance of a stable hemodynamic status in the acute period of ischemic stroke remains an unresolved issue, particularly after revascularization therapies [3, 19, 20]. Of note, dCA plays an important role in the management of cerebral hemodynamics; it can not only prevent hemorrhagic transformation caused by hyperperfusion after MT but also prevent infarct expansion caused by hypoperfusion. Thus, dCA is theoretically important for favorable outcomes in patients after MT. Until recently, there was a lack of dCA data concerning such patients. Tian et al. [21] showed that dCA was bilaterally impaired until 7 days after stroke onset in patients who underwent MT. However, because healthy adults were selected as the control group in that study, it remains unknown whether MT affects dCA when compared to no MT. In the present study, we found significant improvement in dCA after MT, which demonstrated that the damage to the vascular wall by MT surgery was completely offset by the positive effect of recanalization on dCA.

Furthermore, we found that the PD of the affected side within 3 days after MT was an independent predictor of favorable outcomes. The area under the ROC curve for the prediction of favorable outcomes was 0.759, and the best cutoff point was 18.34°, suggesting that reaching this value could be regarded as an acceptable improvement goal in clinical practice. Special attention should be given to patients with a PD of less than 18.34° after MT because these patients have a high probability of an unfavorable outcome. In addition, our results demonstrated that the collateral score was associated with dCA function, suggesting that the collateral reserve capacity is critical in patients undergoing MT.

This study had several limitations. First, to ensure that patients received treatment as early as possible, we did not collect dCA data before MT; therefore, we were unable to compare baseline dCA levels between the two groups. As of now, it remains difficult to overcome this issue because the dCA measurement takes nearly 30 min, meaning that performing dCA tests before MT would significantly delay reperfusion therapy. Prospectively, as technology progresses and dCA testing becomes more advanced, the dCA measurement time may be significantly reduced, which may make it possible to complete dCA measurements without delaying MT. Second, in clinical practice, patients with posterior large vessel occlusion account for a certain proportion of cases, and we also included such patients; however, due to the limited sample size, we were unable to analyze them separately. Further studies focused on patients with posterior circulation stroke should be conducted. Third, the sample size of our study was relatively small, and our findings warrant validation in further large‐scale investigations. Fourth, this observational study only revealed that MT has a positive influence on dCA; however, the underlying mechanisms remain unclear and deserve further exploration in animal experiments. Additionally, interventional studies are needed to investigate whether dCA can be used as an intervention target to improve the prognosis of MT patients.

## 5 Conclusions

MT has a positive effect on dCA in patients in the acute phase of stroke. dCA is a reliable indicator for predicting the prognosis of patients with acute stroke after MT, and it may also be a specific intervention target to improve prognosis in these patients.

## Author Contributions

Y.Y., Z.‐N.G., Y.Q., and M.C. drafted the initial protocol, which was reviewed with critical revision and approval by all authors. Y.Q. and M.C. wrote the first draft of the manuscript. Y.Q., M.C., and P.Z. did the statistical analysis. Y.Q., M.C., Z.‐D.S., H.Z., Y.S., P.‐D.Z., C.L., J.L., K.Z., and J.R. collected data. J.L., J.C., H.J., X.S., and Y.Y. revised the manuscript. Y.Y. and Z.‐N.G. acquired funding. All authors contributed to data acquisition.

## Disclosure

The authors have nothing to report.

## Ethics Statement

The present study was approved by the Ethics Committee of The First Hospital of Jilin University (2016–294).

## Consent

All participants gave written informed consent, and the study was conducted in accordance with the Helsinki declaration.

## Conflicts of Interest

The authors declare no conflicts of interest.

## Data Availability

Data is available from the authors upon request.
